# Features of Peripheral T Cell Remigration into the Thymus

**DOI:** 10.3390/ijms262110391

**Published:** 2025-10-25

**Authors:** Anastasiia A. Kalinina, Ludmila M. Khromykh, Dmitry B. Kazansky

**Affiliations:** N. N. Blokhin National Medical Research Center of Oncology of the Ministry of Health of the Russian Federation, Kashirskoe sh., 24, Moscow 115478, Russia

**Keywords:** thymus, T cell, remigration, intrathymic selection, central tolerance

## Abstract

The thymus, the central organ of T lymphopoiesis, is traditionally thought to exclusively export T cells. However, a great deal of studies has shown that mature peripheral T cells can return to the thymus and remain there. It is acknowledged that both CD4^+^ and CD8^+^ activated T cells can remigrate into the healthy adult thymus and accumulate predominantly in the medulla. In contrast, naïve T cells can actively populate the thymus of neonates and aged animals, potentially supporting the medulla’s functioning. Still, the fate and functions of peripheral T cell remigrants are not fully understood as of today. This review presents experimental findings on peripheral T cell remigration, analyzes phenotypic and traffic features of remigrants, and considers possible effects of backmigration on thymus function.

## 1. Introduction

T cells, one of the key effectors of adaptive immunity, are equipped to recognize foreign antigens via a highly specialized receptor, the T cell receptor (TCR) with a unique structure in each individual clonotype. The estimated theoretical diversity of the TCR repertoire ranges from 10^15^ to 10^20^ clonotypes [1]. It forms during the T cell intrathymic development and undergoes dynamic changes in the periphery during T cell contacts with self and foreign antigens and under the physiological aging of the immune system [2]. The classical paradigm posits that the thymus exclusively exports T cells: T lymphocytes that have matured from bone marrow progenitors exit the thymus to repopulate the periphery [3]. Yet, after activation on the periphery, antigen-specific T cells were shown to migrate into the thymus as a site of infection [4,5,6,7] or inflammation [8]. Importantly, remigrated T cells sustained in the thymus for a long time, strongly responded in secondary immune responses, and, thus, could be considered as tissue-resident memory T cells [4,9,10,11]. Multiple experimental data have since provided evidence that mature peripheral T cells can recirculate back into the thymus even in the absence of active infection processes in this organ, i.e., not just due to effector migration to the localization site of the specific antigen.

This review summarizes findings on mature peripheral T cell remigration into the thymus, characterizes their phenotype and traffic, and addresses the potential effects of such back-migration on thymus function.

## 2. The Thymus: Structure and Functions

The thymus is the central organ of T lymphopoiesis. Its structure and functions are thoroughly covered in many reviews (e.g., [3,12]); therefore, only principal moments will be discussed in this section.

The thymus consists of two lobes, each with a cortex and medulla surrounded by a capsule. The outer cortex contains dense clusters of immature thymocytes and epithelial cells that are involved in T cell maturation. Maturing T cells, macrophages, dendritic cells, and Hassall’s corpuscles (concentrically located epithelial cells) are located in the medulla. The transcription factor autoimmune regulator (AIRE) regulates the expression of various tissue-specific antigens that are presented by molecules of the major histocompatibility complex (MHC) on medullary epithelial cells. Interactions of developing T cells with these MHC-peptide (pMHC) complexes provide effective elimination of potentially autoreactive cells from the repertoire [13,14].

Thymocyte maturation proceeds through several stages and is closely linked to the TCR formation, an αβ-heterodimer in the majority of T cells. TCR generation starts with the gene rearrangement of the β-chain that involves the D (Diversity) and J (Joining) segment joining, followed by the V (Variable) segment joining. This process is mediated by RAG1 and RAG2 recombinases (recombination activating genes). The β-chain gene rearrangement occurs in CD4^−^CD8^−^ double negative (DN) thymocytes that actively proliferate in the subcapsular zone of the thymus. Successful gene rearrangement results in the synthesis of a β-chain capable of pairing with the surrogate α-chain, forming a temporary complex—preTCR. The complexing of preTCR with CD3 provides the signal to a thymocyte to continue its development. In this stage, the simultaneous expression of CD4 and CD8 co-receptors is induced in the thymocyte that becomes the double positive (DP) cell. In the DP thymocytes, the gene rearrangement of the α-chain TCR is initiated, which is also mediated by RAG1/2 recombinases and involves V and J segment joining. A fully functional TCR forms when α-chain gene rearrangements are finished [13,14].

DP thymocytes that are located in the cortex undergo positive selection, which is aimed at selecting TCRs capable of interacting with self-pMHC complexes with sufficient affinity. The range of such affinities has minimal and maximal thresholds that increase with T cell maturation. Thymocytes expressing TCRs with affinity below the minimal threshold die by apoptosis (the so-called death by neglect). Surviving DP thymocytes migrate into the medulla, where they undergo negative selection. It is aimed at eliminating T cells, whose TCRs interact with self-pMHC complexes with an affinity above the maximal threshold. Such potentially autoreactive T cells are eliminated by apoptosis [13]. After the selection, single positive CD8^+^ or CD4^+^ T cells develop and migrate from the thymus to form the peripheral pool.

Importantly, not all thymocytes with high affinity TCRs to self-pMHC complexes die by apoptosis during negative selection. The high affinity interaction of a TCR with the ligand can induce the differentiation of CD4^+^ cells into regulatory T cells (Tregs) that stably express the transcription factor forkhead box protein 3 (FOXP3) [15,16]. As with Treg generation, CD8^+^ T cells, expressing TCRs with high affinity to self-pMHC class I complexes, can avoid apoptosis during the intrathymic development by differentiating into the so-called virtual (or memory-phenotype [17]) memory T cells [18]. Interestingly, we detected peripheral mature CD8^+^ T cells that could effectively interact with self MHC class II molecules. This finding directly indicated that autoreactive T cells could survive negative selection by alternative commitment and acquisition of the CD8 co-receptor instead of the CD4 co-receptor [19]. This mechanism is apparently aimed at maximally preserving and using the productive TCR gene rearrangements formed during intrathymic development. However, in case of its malfunction, this mechanism also creates the risk of developing autoimmune diseases.

## 3. Remigration of Peripheral T Cells into the Thymus

The remigration of mature peripheral T cells into the thymus was mainly studied in experimental mouse and rat models (Table 1). This phenomenon was also observed in pigs and sheep [20,21,22]. Similar populations of mature peripheral T cells were detected in the adult human thymus [11,23].

The permeability of the thymus for peripheral T cells was shown to change with aging [24,25,26,42]. Surh et al. compared the numbers of mature donor Thy1.2 T cells in the thymus of newborn (1–7-day-old) and adult (12-week-old) Thy1.1 recipient mice after the adoptive transfer of lymphocytes from the lymph nodes of adult donors. The dose of the adoptively transferred cells was normalized by the recipient’s body weight. In the newborn thymus, donor T cells comprised 0.14% of the total number of transferred lymphocytes. Migrated mature donor T cells were localized predominantly in the medulla of the newborn thymus and were sustained there for two months following the adoptive transfer. Meanwhile, donor T cells were practically absent in the thymus of adult recipients (0.01–0.02% of the total dose of transferred lymphocytes). This could be due to their preferential migration into lymph nodes, not observed in neonates [24]. In a subsequent study, Hale et al. used transgenic mice with the expression of green fluorescent protein (GFP) under the RAG2 promoter. This reporter model allowed for monitoring recent thymic emigrants (GFP^+^ cells) during their maturation on the periphery, which is accompanied by the gradual loss of the GFP expression [26]. Approximately 1–2% of remigrated mature GFP^−^ T cells were detected in the thymus of young (6-week-old) Rag2p-GFP transgenic mice. The proportion of remigrated mature T cells increased with animal aging and reached 25% in the thymus of mice older than one year [26].

Importantly, GFP^−^ T cells detected in the thymus of young Rag2p-GFP mice had the CD44^high^ phenotype, suggesting that activation of mature T cells on the periphery preceded their remigration into the thymus [26]. Indeed, compared to newborn [24,42] and aged (older than 68 weeks) [26,43] mice, the thymus of adult animals is practically impermeable for non-activated T cells [24,27,28,29,44]. Furthermore, irradiation and injections of hydrocortisone that deplete CD4^+^CD8^+^ DP thymocytes, thus creating niches for repopulation, did not stimulate the traffic of naïve (non-activated) T cells into the adult thymus [45]. According to numerous studies, activated T cells robustly remigrated into the thymus compared to naïve cells [4,5,6,30,31,32,33,34,35]. T cells in the S phase were shown to enter the thymus more actively, although quiescent memory cells can also remigrate [44].

The pathological processes in the thymus markedly stimulate its infiltration with peripheral T cells [4,5,6,8,36,37]. Michie et al. studied AKR mice with a high rate of T cell lymphomas and found that the infiltration of their hyperplastic medulla with peripheral T cells was increased with animal age [36]. Inflammation in the medulla or intrathymic infections also stimulated migration of mature T cells into the thymus [4,5,6,8]. In the latter case, however, the activated antigen-specific peripheral effectors migrated into the thymus as an inflammation site and participated in the local immune response [4,5,6], which resulted in the formation of thymic resident memory cells that provided protection from reinfection [4]. Importantly, inflammation within the thymus medulla can facilitate remigration of not only antigen-specific T cells but also mature T cells with unrelated TCR specificities, as found in the work of Levinson et al. [8]. In their studies, the authors found that some CD4^+^ T cells specific to the α subunit of the acetylcholine receptor (AChRα) can escape negative selection and re-enter the thymus under the inflammatory processes in the medulla. These autoreactive CD4^+^ T cells can recognize AChRα, expressed on myoid cells and thymic epithelial cells, leading to their activation. The activated AChRα-specific CD4^+^ T cells, in turn, can provide help to AChRα-reactive B cells, localized in the thymus, which then produce anti-AChRα antibodies. This sequential mechanism represented the intrathymic pathogenesis of myasthenia gravis [8].

## 4. Features of Remigrating T Cells

Both CD4^+^ and CD8^+^ T cells can remigrate into the thymus [5,10,24,26,27,28,29,35,38,46]. Experiments with the adoptive transfer of the phenotype-sorted mature T cells showed that T cells with the activation phenotype of effectors actively enter the thymus [28,29]. Other studies confirmed that naïve peripheral T cells were detected in a healthy adult thymus only in minor numbers (hundredths of a percent of the thymus cells) [10,26,47]. Meanwhile, mature non-activated (naïve) CD45RB^high^CD44^low^ T cells can actively enter a newborn thymus [24]. Interestingly, naïve T cells migrated into the lymphopenic thymus of adult mice with severe combined immunodeficiency (SCID) as effectively as into the thymus of healthy neonates. Studies showed that remigrated T cells contributed to the regeneration of the medullary thymic epithelium in SCID mice [37].

The permeability of the thymus to non-activated peripheral T cells also increases with aging [26]. Adoptive transfer of naïve CD44^low^CD62L^high^ CD8^+^ T cells into young (8–10-week-old) and aged (70–76-week-old) Rag2p-GFP mice demonstrated that donor GFP^−^ T cells were practically absent in the thymus of young recipients but actively accumulated in the thymus of aged mice, while preserving the phenotype of naïve cells [26].

**Figure 1:**
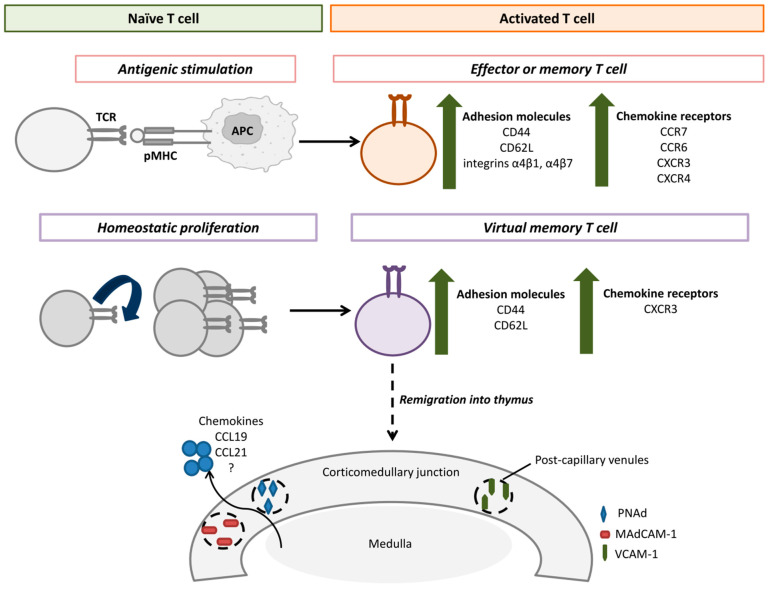
Activated peripheral T cells can remigrate into the thymus. After antigenic stimulation or homeostatic proliferation, T cells acquire the activated phenotype, characterized by the upregulation of adhesion molecules and chemokine receptors. This could facilitate the migration of peripheral T cells into the corticomedullary junction of the thymus via post-capillary venules that express adhesion molecules PNAd, MadCAM-1, and VCAM-1. Chemokines CCL19, CCL21, and others, produced by medullary thymic epithelial cells, could presumably regulate traffic of activated T cells into the thymus. TCR—T cell receptor, APC—antigen-presenting cell, pMHC—MHC-peptide complex, PNAd—Peripheral node addressin, MAdCAM-1—Mucosal vascular addressin cell adhesion molecule 1, VCAM-1—Vascular cell adhesion molecule 1.

According to the general consensus, the acquisition of an activated phenotype by a T cell is a critical prerequisite for their remigration into the adult thymus [35,44,45]. Antigenic stimulation on the periphery [4,5,6,30,31] or in vitro activation [23] was shown to significantly enhance T cell remigration. T cell activation is also associated with changes in the expression profile of adhesion molecules and chemokine receptors, which significantly impact their migration [46] and, in particular, may mediate their traffic into the thymus (Figure 1). Interestingly, homeostatic proliferation under the lymphopenia also stimulated remigration of mature peripheral T cells into the thymus even without antigenic stimulation [27,33,34,35]. During homeostatic proliferation, T cells acquired the activation phenotype of memory cells CD44^high^CD62L^high^, which is why they were called virtual memory cells (memory-phenotype cells) [17,48,49]. Presumably, such T cells can remigrate into the thymus due to their phenotypic features, similar to those of antigen-stimulated effectors [50] (Figure 1).

Studies of Tregs remigration are of particular note [23,39,51,52]. Using transgenic mice with GFP expression under the RAG2 promoter and Thy1.1 expression under the FOXP3 promoter, Thiault et al. showed that GFP^−^ cells could be detected in the thymus of these animals, and their proportion within CD4^+^CD8^−^Thy-1.1^+^ Tregs increased from 10% at the age of 10 weeks to 90% at the age of 50 weeks [23]. The transcriptome analysis revealed that these GFP^−^ Tregs differentiated from the spleen GFP^+^ Tregs and actively expressed genes of effector molecules, characteristic of peripheral Tregs (e.g., Tgfb1, Tgfb2, Il10, Ctla4, etc.). Furthermore, changes in the repertoire of the β-chain TCR variable segments (Vβ) of peripheral T cells (the decreased proportion of Vβ5^+^ cells due to their partial depletion and the increased proportions of Vβ6^+^ and Vβ11^+^ T cells due to their polyclonal proliferation) were completely reflected in the Vβ repertoire of GFP^−^ Tregs, localized in the thymus [23]. Taken together, the findings of this study confirmed that peripheral Tregs can remigrate to the thymus. Interestingly, this effect was also observed in the human thymus: cells that did not express CD31 (the marker of developing thymocytes and early thymic emigrants) were detected in the thymic population of mature CD4^+^ T cells. CD31^−^CD4^+^ T cells expressed the high level of FOXP3, the transcription factor T-bet, and inducible T cell costimulator (ICOS) that reflected their differentiated phenotype and marked the peripheral origin [23]. The traffic of peripheral Tregs into the thymus does not contradict the concept that predominantly activated mature T cells can remigrate, as Tregs have the activated phenotype CD44^high^CD25^high^ [23,50].

## 5. The Pathways of Peripheral T Cell Remigration

Numerous studies demonstrated that mature T cells are located mainly in the thymus medulla (Table 1), entering via post-capillary venules in the corticomedullary junction [46], whereas peripheral Tregs can populate both the cortex and the medulla [23]. The initial experimental data on the presumed mechanisms of T cell remigration were obtained in the model of AKR mice [7]. Michie et al. found that hyperplastic changes in the thymus medulla of these mice were accompanied by increased numbers of high endothelial venules that express peripheral node addressin (PNAd) and mucosal vascular addressin cell adhesion molecule 1 (MAdCAM-1). Functional in vitro and in vivo tests revealed that peripheral T cells populate the thymus via interactions between integrin α4β7 with MAdCAM-1 and L-selectin (CD62L) with PNAd [7]. A study with healthy adult rats showed that activated T cells remigrate into the thymus via interactions of integrin α4β1 with vascular cell adhesion molecule 1 (VCAM-1) [28]. Different adhesion molecules, including CD44, CD62L, and integrins α4β1 and α4β7, are upregulated in activated effectors and memory cells [47] (Figure 1), presumably allowing them to enter the thymus (whose capillaries normally express a low level of addressins) more successfully than naive cells [42].

Yet, several studies indicated that peripheral T cells found in the thymus did not express CD62L [4,10,11,23]. This adhesion molecule could be important for T cells to enter the thymus [7], then its expression subsequently decreases. There is a parallel here with resident memory cells, which have also been shown to display low levels of CD62L [51]. The expression of the characteristic markers CD103 and CD69 [4,11,23,51] and the long-term sustention without re-export [4,10,11,24,27,32] make remigrated T cells even more similar to resident memory cells.

Today, little is known about cytokines that regulate peripheral T cell remigration into the thymus. It was proposed that chemokines CCL19 and CCL21, actively produced in the medulla, could control this process [45]. Naïve T cells and CD44^high^CD62L^high^ central memory cells actively express CCR7, the receptor of these chemokines [47,52]. Hence, it can mediate their traffic into the thymus. However, CCR7 was shown to interfere with Tregs remigration, and peripheral Tregs, detected in the thymus, expressed CCR6 but not CCR7 [40]. CCR6 is expressed on effectors and memory cells but not naïve T cells [53]. Remigration of peripheral Tregs could also be mediated by the chemokine receptor CXCR4. AMD3100, the CXCR4 inhibitor, was shown to decrease Tregs traffic into the thymus without affecting their migration into the spleen [23]. Of particular note, mature CCR7^−^ CD8^+^ T cells were detected in the healthy adult thymus [11]. These cells expressed integrin α4β7 and the chemokine receptor CXCR3 [11], characteristic of activated CD8^+^ cells [47]. Interestingly, virtual memory cells that form during homeostatic proliferation also expressed CXCR3 [54,55]. As highlighted above, virtual memory cells are capable of remigration; thus, it seems plausible that CXCR3 can regulate the traffic of mature T cells into the thymus.

## 6. Functions of Remigrated Mature T Cells in the Thymus

Early research found that the thymus can be a repository of antigen-specific effectors and memory cells [56,57], which is in line with the long-term persistence of peripheral T cells in this organ [4,10,11,24,27,32]. However, the functional consequences of T cell remigration, both for remigrants and for the intrathymic processes, were unknown.

Chau et al. found peripheral T cells in the thymus of mice with allogeneic heart transplants. These mature T cells were alloreactive CD44^high^ cells, activated on the periphery by transplant antigens. Interestingly, some remigrated T cells died by apoptosis in the medulla of the recipient’s thymus. This was proposed as a mechanism of the tolerance induction to the allotransplant. The authors further suggested that induction of apoptosis in remigrated T lymphocytes could be a more general mechanism for maintaining the homeostasis of the peripheral T cell pool, ensuring the elimination of excess antigen-specific clones during the contraction of the immune response [31].

Considering that remigrated mature T cells localize predominantly in the thymic medulla, they could influence its functioning. As highlighted above, developing thymocytes undergo negative selection in the medulla. Several studies indicated that remigrated peripheral T cells participated in the induction of central tolerance [25,30,31,33,41]. Webb and Sprent used mouse lines that differed in superantigens of mouse mammary tumor virus (MMTV) (minor lymphocyte stimulating (Mls) antigens). They showed that the adoptive transfer of Mls^a^ T cells into newborn Mls^b^ recipients induced tolerance to Mls^a^ antigens. This effect was due to the migration of donor T cells into the recipient’s thymus, where they presented Mls^a^ as a tolerogen to developing thymocytes. This resulted in the depletion of T cells with the Vβ6 β-chain TCR V segment that precisely respond to Mls^a^ [25]. Several other studies used transgenic CBK mice that expressed the MHC class I molecule H-2K^b^ on the genetic background of CBA/CaJ mice [33,41]. In the developed adoptive transfer system, it was shown that T cells from CBK donors localized in the thymus of CBA/CaJ recipients and presented the allogeneic MHC class I molecule to thymocytes. This induced negative selection of developing alloreactive CD8^+^ T cells, ensuring long-term engraftment of CBK skin grafts [33,41].

Of note, the efficacy of negative selection declined in the thymus of newborn and aged mice because of the medullary epithelial dysfunctions [58,59,60]. As pointed out above, peripheral T cells, including Tregs, can actively enter the thymus of animals of these ages [23,24,25,26,43]. In this case, the enhanced traffic of mature T cells can presumably support the functional activity of the medulla.

However, the work of Edelmann et al. revealed that remigrated T cells could interfere with central tolerance induction [34]. The authors used transgenic RIP-mOVA and OT-1 mice, both on the genetic background of the C57BL/6 line. The RIP-mOVA mice expressed membrane-bound chicken ovalbumin (OVA) in β cells and medullary thymic epithelial cells. In OT-1 mice, CD8^+^ T cells expressed the receptor specific to the OVA peptide, presented in the complex of the MHC class I molecule H2-K^b^. The adoptive transfer of CD8^+^ OT-1 T cells into the RIP-mOVA recipients induced the accumulation of activated donor T cells in the recipient’s thymus. These donor T cells eliminated the dendritic cells and medullary epithelial cells that expressed the OVA peptide, impeding negative selection and leading to the generation of autoreactive OVA-specific CD8^+^ T cells in RIP-mOVA mice [34].

To uncover possible functions of remigrated T cells in the thymus, Kirberg et al. generated OT-1 mice with double knockouts for Rag2 and H-2K^b^. Since positive selection of the OT-1 receptor requires the MHC class I molecule H-2K^b^, the thymocyte development halted in the DP stage in these mice because of the absence of the selecting ligand [35]. The adoptive transfer of syngeneic H-2K^b^-positive T cells into double knockout OT-1 mice resulted in the generation of OT-1 CD8^+^ T cells in the thymus of recipients. In this experimental system, precisely donor T cells expressed the H-2K^b^ molecule that controlled positive selection of OT-1 cells. Donor H-2K^b^-positive T cells migrated into the medulla and, to a lesser extent, the cortex of the recipient’s thymus and directly presented the selecting ligand to developing thymocytes [35].

Taken together, T cell remigration can influence the intrathymic selection and, consequently, TCR repertoire formation and potentially contribute to the maintenance of the peripheral pool homeostasis. Interestingly, Tregs remigration can also be seen as a possible strategy for sustaining peripheral pool homeostasis, as remigrated Tregs prevented interleukin-2-dependent de novo development of Tregs in the thymus [23].

## 7. A Proposed Hypothesis of TCR Editing in Remigrated T Cells

**Figure 2:**
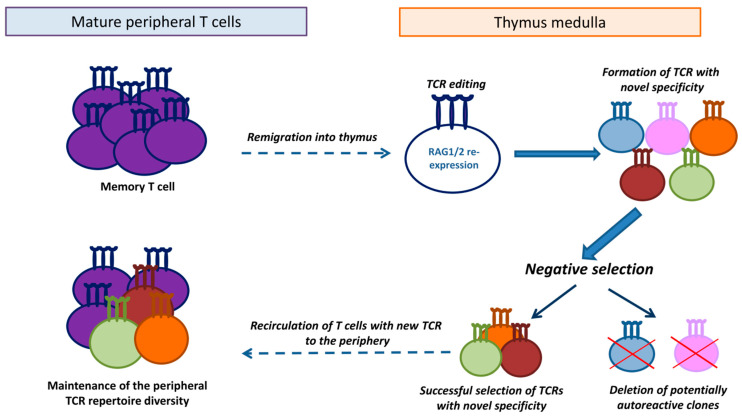
A hypothesized function of T cell remigration in sustaining the homeostasis of the peripheral pool. Memory T cells generated during the immune response remigrate into the medullary zone of the thymus, where re-expression of RAG1/2 recombinases and T cell receptor (TCR) editing can occur. This results in the development of TCRs with new specificity followed by selection on MHC-peptide complexes presented by medullary thymic epithelial cells. Negative selection eliminates potentially autoreactive clones, and T cells with edited TCRs that successfully pass negative selection recirculate into the periphery, thus enriching the peripheral TCR repertoire.

In our studies, we found that allospecific memory cells could enter the thymus. These cells were cortisone-resistant and robustly responded in the secondary immune response to the specific (immunizing) antigen [32]. Since the thymus provides the microenvironment for the RAG1/2 recombinase expression and selection of new receptors, we hypothesized that α/β-chain TCR editing could be initiated in remigrated memory T cells, so receptors with new specificities could be generated. Such novel TCRs could purportedly undergo selection on the medullary thymic epithelial cells, and after elimination of potentially autoreactive clones, mature T cells with the edited TCRs could exit the thymus and replenish the peripheral pool (Figure 2). It seems plausible that such a suggested mechanism can eliminate the excess of memory cell clones with similar specificities, formed during the immune response [61].

Studies of secondary allogeneic immune responses in B6.129S2-Tcra^tm1Mom^/J mice, heterozygotes for the α-chain TCR knockout, partially corroborated this hypothesis. In these mice with limited α-chain gene rearrangements, significantly enhanced secondary responses were detected compared to the wild-type control animals [32]. Hence, remigration of memory T cells into the thymus could be aimed at maintaining the diversity of the peripheral T cell repertoire by editing their TCRs and generating receptors with novel specificities (Figure 2).

Currently, the proposed theory is merely a hypothesis. RAG1/2 re-expression in remigrated antigen-specific memory T cells remains to be confirmed. Furthermore, changes in the TCR repertoire of memory T cells formed in the periphery and re-entering the thymus must be tracked (e.g., by NGS) to testify that TCR editing occurs in mature T cells upon their remigration.

## 8. Conclusions

At present, studies have gathered strong experimental evidence regarding peripheral T cell remigration into the thymus. However, the functional effects of such remigration have not been fully studied. The proposed theories on the role of remigrated mature T cells in intrathymic selection and the maintenance of the peripheral homeostasis necessitate further experimental validation. Identifying the fate of remigrant T cells will shed light on new functions of the thymus. Furthermore, understanding what changes such cells undergo may have practical implications.

Particularly, a better understanding of this phenomenon could change the protocols of adoptive cell therapy, an important strategy for cancer treatment. The production of the therapeutic T cell products involves the activation of autologous T cells in vitro, their gene modification with chimeric antigen receptors (CAR-T) or TCR specific to tumor antigens, followed by infusion into the patient. Chemo- and/or radiotherapy frequently precedes the adoptive transfer, thus, the patient’s immune system is often suppressed and lymphopenic. As highlighted in this review, homeostatic proliferation of T cells under lymphopenia facilitates their remigration into the thymus. Hence, it seems plausible that infused CAR-T or TCR-T cells can enter the patient’s thymus and either participate in the selection of developing thymocytes or undergo negative selection.

As suggested by Edelmann et al. based on their findings [34], remigrated therapeutic CAR- or TCR-T cells could specifically target thymic dendritic cells or thymic epithelial cells that express tumor-associated (i.e., self) antigens. Such intervention into the process of central tolerance induction could potentially result in the generation of autoreactive T cell clones with specificity to tumor-associated antigens [34]. This could potentially enhance the host anti-tumor immune response by expanding the TCR repertoire with novel high-affinity receptors specific to tumor-associated antigens. Although this idea was posed nearly a decade ago, evidence has yet to be provided in support of it, and direct research into the idea is of immense interest and practical significance. Tracking the traffic of CAR- or TCR-T cells into the thymus and elucidating their contribution to the intrathymic processes suggest intriguing future directions in the optimization of adoptive cell therapy.

## Figures and Tables

**Table 1:** Research findings on the remigration of mature peripheral T cells into the thymus.

Animals	Experimental Setup	Main Findings	Reference
Mice	LCMV model	Virus-specific T cells were activated on the periphery and migrated into the thymus medulla, where they persisted over one year and possessed features of resident memory cells	[4]
Mice	Mycobacterial infection (*M. tuberculosis* and *M. avium*)	Activated on the periphery mature antigen-specific T cells migrated into the thymus as the site of infection	[5]
Mice	Adoptive transfer of syngeneic CD8^+^ T cells specific to LCMV antigens	Activated virus-specific donor CD8^+^ T cells were detected in the thymus medulla of LCMV-infected recipients	[6]
Mice	Adoptive transfer of CFSE-labeled CD4^+^ T cells specific to an influenza hemagglutinin peptide into mice that were preliminarily immunized with β-galactosidase (β-gal) and intrathymically injected with β-gal-encoded MLV-based vectors	Induced inflammation in the thymus medulla facilitated remigration of mature CD4^+^ T cells with unrelated specificities. Remigrated peripheral CD4^+^ T cells that escaped the negative selection could contribute to the intrathymic pathogenesis of myasthenia gravis	[8]
Thy-1 congenic mice	Adoptive transfer of lymphocytes from the lymph nodes	Mature donor T cells accumulated in the medulla of the recipient’s thymus and possessed features of resident memory cells	[10]
Pigs	Adoptive transfer of CD45-congenic lymphocytes	Donor T cells accumulated predominantly in the corticomedullary junction of the thymus 4 h following the adoptive transfer	[22]
Rag-GFPFoxp3-Thy-1.1 transgenic mice	Migration in situ of peripheral Tregs. Adoptive transfer of in vitro-activated Tregs from Foxp3-Thy-1.1 transgenic mice into wild type recipients	The intensity of accumulation of mature peripheral GFP^−^ Tregs in the thymus grew with age. Remigrated Tregs suppressed interleukin 2-dependent de novo differentiation of Tregs in the thymus	[23]
Thy-1 congenic mice	Adoptive transfer of syngeneic lymphocytes from lymph nodes into 1-day- and 12-week-old recipients	Mature naïve T cells cannot remigrate into the thymus of adult mice (12-week-old) but actively accumulate in the medulla of newborns	[24]
Mouse lines with differences in MMTV superantigens	Adoptive transfer of spleen T cells	Mature T cells remigrated into the thymus of newborn mice and mediated the formation of central tolerance to Mls^a^ superantigens	[25]
Rag2p-GFP transgenic mice	Migration in situ of peripheral T cells. Adoptive transfer of syngeneic T cells into Rag2p-GFP mice	Activated T cells can remigrate into the thymus of young mice. Mature T cells can remigrate into the thymus of aged mice without preliminary activation on the periphery	[26]
Thy-1 congenic mice	Adoptive transfer of naïve T cells and activated T blasts into irradiated and non-irradiated recipients	Naïve donor T cells rarely migrated into the recipient’s thymus. Irradiation or injections of hydrocortisone into the recipient did not stimulate migration of naïve donor T cells into the recipient’s thymus. Activated T cells actively migrated into the thymus and persisted in the medulla for one month. Irradiation of the recipient before the adoptive transfer enhanced migration of donor activated T cells into the recipient’s thymus	[27]
Rats	Adoptive transfer of syngeneic T cells	Predominant remigration of T cells with the phenotype of activated effectors. Remigration of peripheral T cells into the thymus is mediated by interactions of integrin α4 and VCAM-1	[28]
Rats	Adoptive transfer of syngeneic T cells from the spleen and lungs	Predominant migration of antigen-primed CD4^+^ T cells into the thymus medulla	[29]
Rats	Adoptive transfer of radioactively labeled syngeneic alloreactive T cells	Activated T cells migrated into the thymus and were engaged in the induction of central tolerance	[30]
Mice with allogeneic heart transplant	Migration in situ of peripheral T cells in the recipient with allotransplant. Adoptive transfer of CFSE-labeled T cells into the recipient with allotransplant	Activated alloreactive T cells migrated only into the thymus of recipients with allotransplants. Remigrated alloreactive T cells died by apoptosis in the thymus medulla	[31]
Mice	Immunization of C57BL/6 (H-2^b^) with allogeneic mastocytoma P815 (H-2^d^)	Alloreactive memory T cells were detected in the thymus of immunized mice, responded in the secondary immune response in vitro, and were hydrocortisone-resistant	[32]
Mice	Adoptive transfer of allogeneic T cells into lethally irradiated recipients	Mature donor T cells could migrate into the recipient’s thymus only after homeostatic proliferation on the periphery. Migrated T cells were engaged in the formation of central tolerance	[33]
Mice	Adoptive transfer of syngeneic OT-I CD8^+^ T cells into C57BL/6 or RIP-mOVA transgenic mice	Remigration of peripheral T cells was enhanced after homeostatic proliferation under lymphopenia. Donor OT-1 T cells eliminated dendritic cells and medullary epithelial cells that express the OVA peptide in the recipient RIP-mOVA thymus. Consequently, autoreactive OVA-specific CD8^+^ T cells were generated in RIP-mOVA recipients	[34]
Mice	Adoptive transfer of H-2Kb^+^ T cells into H-2Kb^−^ OT-1 transgenic mice	Donor T cells were located in the medulla and, to a lesser extent, the cortex of the recipient’s thymus and participated in positive selection of developing thymocytes by directly presenting the alloantigen (the H-2K^b^ molecule)	[35]
AKR mice	Adoptive transfer of syngeneic lymphocytes from lymph nodes	Enhanced migration of peripheral T cells into the hyperplastic thymus of AKR mice via interactions of the homing receptors L-selectin and integrin α4β7 with addressins PNAd and MAdCAM-1, respectively	[36]
Thy-1 congenic mice	Adoptive transfer of mature T cells into SCID mice	Peripheral T cells, including with the phenotype of naïve cells, actively migrated into the thymus of adult SCID mice. Donor T cells accumulated in the medulla of the SCID thymus and regulated the functions of medullary epithelial cells	[37]
Mice	Parabiosis between C57BL/6-GFP and pTα knockout mice and between wild type Ly5.1^+^ mice and Ly5.2^+^ pTα knockout mice	Donor CD4^+^ and CD8^+^ T cells accumulated in the thymus of lymphopenic pTα knockout mice	[38]
CD45 congenic mice	Adoptive transfer of CD45.1^+^ CD4^+^ T cells into irradiated CD45.2^+^ wild type mice or non-irradiated CD45.2^+^ RAG1 knockout mice	Donor Tregs were detected in the thymus of adult recipients. The numbers of donor Tregs in the thymus positively correlated with their numbers in the recipient’s spleen	[39]
Rag2p-GFP and Rag2pGFP/ Foxp3RFP transgenic mice	Migration in situ of peripheral Tregs	Peripheral GFP^−^ Tregs accumulated in the thymus and had the phenotype CCR7^−^CCR6^+^. CCR7 prevented remigration of Tregs into the thymus	[40]
Mice	Adoptive transfer of allogeneic T cells into lethally irradiated recipients	Activated donor T cells migrated into the thymus of irradiated recipients and contributed to the induction of central tolerance	[41]

## Data Availability

No new data were created or analyzed in this study. Data sharing is not applicable to this article.

## Abbreviations

TCR—T cell receptor, APC—antigen-presenting cell, pMHC—MHC-peptide complex, MLV—murine leukemia virus, AChRα—α subunit of the acetylcholine receptor, PNaD—Peripheral node addressin, MAdCAM-1—Mucosal vascular addressin cell adhesion molecule 1, VCAM-1—Vascular cell adhesion molecule 1, SCID—severe combined immunodeficiency, LCMV—Lymphocytic choriomeningitis virus, AIRE—autoimmune regulator, DN—double negative thymocyte, DP—double positive thymocyte, FOXP3—forkhead box protein 3, Treg—regulatory T cell, MMTV—Mouse mammary tumor virus, CCR—CC chemokine receptor, CCL—Chemokine (C-C motif) ligand, CXCR—C-X-C chemokine receptor, GFP—green fluorescent protein, Tbet—T-box expressed in T cells, ICOS—inducible T cells costimulator, Mls—minor lymphocyte stimulating antigen, OVA—ovalbumin.
